# Utility of QuantiFERON TB gold test in a south Indian patient population of ocular inflammation

**DOI:** 10.4103/0301-4738.57147

**Published:** 2009

**Authors:** Kalpana Babu, Vidya Satish, S Satish, D K SubbaKrishna, Mariamma Philips Abraham, Krishna R Murthy

**Affiliations:** Vittala International Institute of Ophthalmology & Prabha Eye Clinic And Research Center, India; 1 Santosh Diagnostic centre, India; 2 Department of Biostatistics, National Institute of Mental Health Sciences, Bangalore, India

**Keywords:** Interferon-γ release assays, intraocular tuberculosis, QuantiFERON TB gold test

## Abstract

**Aim:**

To study the utility of interferon-γ release assays (QuantiFERON TB gold test) in a south Indian patient population of intraocular inflammation.

**Design:**

Evaluation of a diagnostic test- a pilot study from January 2007 to October 2008.

**Materials and Methods:**

QuantiFERON TB gold test was performed on the following groups of patients following an informed consent. Group A included healthy volunteers without any exposure to tuberculosis (TB) or past history of TB (n=22). Group B included patients with active systemic TB diagnosed by the demonstration of acid-fast bacilli or by the histopathology finding of caseation with granuloma formation from the sputum, lymph node, skin or intestinal biopsies (n=26). Group C included patients with uveitis of known etiologies other than intraocular TB without any history of exposure to active TB (n=21). Group D included patients with a diagnosis of presumed intraocular TB, who responded to antitubercular therapy by decreased or no recurrences following treatment and with a minimum of nine months follow-up following initiation of antitubercular therapy (n=39).

**Results:**

The sensitivity and specificity of the QuantiFERON TB gold test to pick up active systemic TB was 58% and 77% respectively. The sensitivity and specificity of the QuantiFERON TB gold test to pickup intraocular TB was 82% and 76% respectively.

**Conclusions:**

QuantiFERON TB gold test alone may not be specific for intraocular TB. The significance of this test in a case scenario needs to be interpreted with clinical presentation and other evidences for intraocular TB.

The diagnosis of intraocular tuberculosis (TB) is difficult due to the large variations in clinical presentations, lack of uniformity in diagnostic criteria, and the low yield of organisms from the eye.[1] One has to rely on the clinical presentations, positive purified protein derivative (PPD) test reaction and molecular diagnostic techniques to make a diagnosis of presumed intraocular TB. Newer diagnostic methods are challenging. Their utility in the Indian scenario, where TB is endemic, is still unanswered.[2,3] Interferon gamma release assays are *in vitro* assays, which measure the interferon-γ (IFN-γ) released by sensitized T cells after stimulation by the *Mycobacterium tuberculosis* antigen. The *Mycobacterium tuberculosis* antigens used are early secretory antigenic target-6 (ESAT-6) and culture filtrate protein-10 (CFP-10). All *M.tuberculosis* and pathogenic *M.bovis* strains secrete these antigens. As they are absent from all *Bacille Calmette-Guerin* (BCG) vaccine strains and from commonly encountered non-tuberculous mycobacterium except *M. Kansasii, M. Szulgai and M. Marinum*, it is more specific for *M. Tuberculosis* than tests that use tuberculin PPD as the antigen.[4] The aim of this study was to study the utility of the new generation interferon-γ release assays (QuantiFERON TB gold test manufactured by Cellestis Limited, Carnegie, Victoria, Australia) in a south Indian patient population with ocular inflammation.

## Materials and Methods

This was a pilot study from January 2007 to October 2008. QuantiFERON TB gold test was performed on the following groups of patients with an informed consent: Group A included healthy volunteers without any history of exposure to active TB or past history of active TB (n=22). Group B included patients with active systemic TB diagnosed by the demonstration of acid-fast bacilli or by the histopathology finding of caseation with granuloma formation from the sputum, lymph node, skin or intestinal biopsies (n=26). Group C included patients with uveitis of known etiologies other than intraocular TB without any history of exposure to active TB (n=21). Group D included patients with a diagnosis of presumed intraocular TB as per the suggested intraocular TB guidelines^1^ (Annexure 1) and who responded to antitubercular therapy by improvement or no recurrences following treatment, with a minimum of nine months follow-up following initiation of antitubercular therapy (n=39).

This study was approved by our institutional review board and the data was analyzed using the SPSS-15 (SPSS Inc., Chicago, Illinois) statistical package. The diagnosis of presumed intraocular TB was made as per the guidelines for intraocular TB proposed by Gupta *et al*.[1] The four-drug regimen (isoniazid, rifampicin, ethambutol, pyrazinamide) was the standard regimen of antitubercular therapy given in all Group D patients. The duration of treatment was a minimum of nine months. The groups were age and gender-matched. History of prior BCG vaccination could not be obtained in all cases due to the inability to recall by the patient or absence of the BCG scar. Results of the PPD test obtained as a part of the routine investigations in uveitis were retrieved from both Group C and Group D patients. An induration of ≥15 mm with or without bleb formation was taken as positive in an immunocompetent patient.

The kit used was QuantiFERON TB gold (QFT-G, manufactured by Cellestis Limited, Carnegie, Victoria, Australia). Aliquots of heparinised whole blood were incubated with the test antigens for 16-24 h. The blood was incubated with the test antigens ≤ 12 h after collection. Test kits included two mixtures of synthetic peptides representing ESAT-6 and CFP-10 as test antigens, phytohemagglutinin (a mitogen used as a positive assay control) and saline (used as a nil sample to measure the background level of IFN-γ). The amount of IFN-γ released is determined by subtracting the amount in the nil from the amount in the ESAT-6 and CFP-10, or mitogen-stimulated plasma. QFT-G test results can be calculated by using the software provided by the manufacturer.

## Results

In Group A, five out of 22 cases (23%) were positive for the test. In Group B, 15 out of 26 (58%) cases were positive for the test. Group B had 14 cases of cervical lymphadenitis (six out of 14 cases were positive), six cases of pulmonary TB (four out of six cases were positive); three cases of TB of skin (all were positive), two cases of intestinal TB (all were positive) and one case of military TB (negative for the test). Comparing Groups A and B, the sensitivity of the test to pick up active systemic TB was 58% while the specificity of this test for active systemic TB was 77%.

In Group C, there were seven cases of HLA B27-related anterior uveitis (four out of seven cases were positive for the test), five cases each of sarcoidosis and Vogt-Koyanagi-Harada disease with panuveitis (none were positive for the test), one case of fungal endophthalmitis (positive for the test), one case each of toxoplasma retinochoroiditis, Fuchs heterochromic iridocyclitis and Behcets disease (none were positive for the test). Overall, five out of the 21 cases (24%) were positive for the test. In addition, we looked at the PPD positivity in these cases which was positive in two cases.

Group D included three cases of granulomatous anterior uveitis (all were positive for the test), eight cases of nongranulomatous anterior uveitis (seven out of eight cases were positive for the test), 10 cases of intermediate uveitis (nine out of 10 cases were positive for the test), three cases of choroidal granuloma (one out of the three cases was positive for the test), nine cases of granulomatous panuveitis (all were positive for the test), two cases of scleritis (all were positive for the test), four cases of retinal vasculitis (one out of four cases was positive for the test). Overall 32 out of 39 cases (82%) were positive for the QuantiFERON TB gold test. In addition, we looked at the PPD positivity in these cases which was positive in 36 cases (92%) [Table 1]. Comparing groups C and D, the sensitivity of the QuantiFERON TB gold test to pick up intraocular TB was 82% while the specificity of the QuantiFERON TB gold test in intraocular TB was 76%.

**Table 1:** Group D showing the variations in clinical presentations in intraocular tuberculosis, their QuantiFERON TB gold and PPD positivity

Type of uveitis	Total number (n)	QuantiFERON TB gold positivity (n)	PPD positivity (n)	Number positive for both tests (n)	Number positive for only a single test (n)
Granulomatous anterior uveitis	3	3	2	2	1
Nongranulomatous anterior uveitis	8	7	6	5	3
Intermediate uveitis	10	9	10	9	1
Posterior uveitis (choroidal granulomas)	3	1	3	1	2
Panuveitis	9	8	9	8	1
Retinal vasculitis	4	1	4	1	3
Scleritis	2	2	2	2	0

Purified protein derivative (PPD) positivity: 36 out of 39 cases (92%), QuantiFERON TB gold positivity: 32 out of 39 cases (82%)

The number of cases in Groups C and D who were both PPD test and QuantiFERON TB gold tests positive and who had intraocular TB was 29 while the number of cases who were both PPD test and QuantiFERON TB gold tests positive and who did not have intraocular TB was one. Taking only Groups C and D into consideration, the sensitivity of a combination of positive PPD and QuantiFERON TB gold tests in picking up a case of intraocular TB was 74% and the specificity of the combination of the above tests in intraocular TB was 95%.

## Discussion

In India, where tuberculosis is an endemic disease, immune-based tests do not directly detect *M. tuberculosis*. They merely indicate a cellular immune response to recent or remote sensitization with the antigen.[5–7] As on date, we do not have many studies which indicate the sensitivity and specificity of this test in active systemic TB and intraocular TB.[2,3] This is a pilot study primarily to determine the above. From our data, where the sensitivity and the specificity of this test to pick up active systemic TB is 58% and 77% respectively, a negative IFN-γ release assay result would not conclusively rule out active disease in an individual suspected to have active systemic TB. Given the lack of gold standard in presumed intraocular TB, the sensitivity and specificity for active TB may not translate into accuracy for intraocular TB. The utility of a positive PPD test or the Mantoux test has always been controversial in an Indian scenario in view of the increased number of false positives. However, recent studies quote the importance of a Mantoux test in patients with uveitis.[8] The purpose of this study was to assess the utility of the QuantiFERON TB gold test in strengthening our diagnosis of presumed ocular TB. From our data, we may infer that QuantiFERON TB gold test alone may not be of much value in the diagnosis of presumed ocular TB. From our data we may speculate that a combination of positive PPD test and QuantiFERON TB gold test in addition to the clinical presentation may be more useful in picking up ocular TB.

## Conclusions

From our data, we may infer that QuantiFERON TB gold test alone may not be of much value in the diagnosis of presumed ocular TB. Probably a combination of a positive PPD test and QuantiFERON TB gold test in addition to the clinical presentation may be more useful in picking up ocular TB. Larger longitudinal studies with clinical outcomes and the effect of treatment on the test results are required in the future.

## Figures and Tables

**Table:** 

**Clinical Signs**
Anterior uveitis: Granulomatous, nongranulomatous, iris nodules, ciliary body tuberculoma Intermediate uveitis: Granulomatous, nongranulomatous with organizing exudates in the parsplana/ peripheral uvea Posterior and panuveitis: Choroidal tubercle, Choroidal tuberculoma, Subretinal abscess, Serpiginous-like choroiditis Retinitis and retinal vasculitis Neuroretinitis and optic neuropathy Endophthalmitis and panophthalmitis Eales disease is considered by some to reflect tuberculous infection/hypersensitivity.
**Ocular Investigations**
Demonstration of AFB by microscope or culture of *M. tuberculosis* from the ocular fluids Positive polymerase chain reaction from ocular fluids for IS 6110 or other conserved sequences in *M. tuberculosis* genome.
**Systemic Investigations**
Positive Mantoux reaction Evidence of healed or active tubercular lesion on radiography of the chest Evidence of confirmed active extrapulmonary tuberculosis (either by microscopic examination or by culture of the affected tissue for *M. tuberculosis*).
**Exclusion of Other Uveitis Entities**
In the geographic regions where tuberculosis is low in incidence, other causes of uveitis must be excluded by various laboratory investigations including serology for syphilis, toxoplasmosis and others.
**Therapeutic Test**
A positive response to four-drug ATT (isoniazid, rifampicin, ethambutol, and pyrazinamide) over a period of four to six weeks. Therapeutic trial with single-drug isoniazid should be avoided due to risk of development of resistance. It is important to refer such a patient to a TB expert who can initiate and monitor the treatment. The therapeutic response to ATT in the eye should, however, be evaluated by the ophthalmologist.
Any one or more of the clinical signs listed under subsequent Section 1 in combination with any of the positive tests under Section 2 could be considered a confirmed (definitive) case of intraocular tuberculosis.
Any one or more of the clinical signs listed under Section 1 in combination with any of the positive tests under Section 3 or a positive therapeutic trial Section 5 in combination with 4 could be considered presumed ocular tuberculosis and referred to a TB specialist to initiate a full course of ATT.
